# Aortic robotic surgery in Ireland: infrastructure without implementation – Is now the time to act?

**DOI:** 10.1007/s11845-026-04377-0

**Published:** 2026-04-18

**Authors:** Morgan T. McLoughlin, Gergely Gosi

**Affiliations:** Department of Vascular Surgery, University Hospital Waterford, Dunmore Road, Waterford, Ireland

**Keywords:** Aortic surgery, Robotic-assisted surgery, Vascular innovation, Ireland, Surgical governance, Minimally invasive surgery, Advocacy

## Abstract

**Background:**

Robotic-assisted surgery is established in several specialties, but its vascular surgery application remains limited. We assessed Irish infrastructural readiness and governance to inform a national feasibility programme.

**Methods:**

A structured environmental scan of Irish tertiary centres was performed. Public sources (hospital/health-service websites, reports, press releases, procurement notices, training pages, professional societies, and newspaper articles) were reviewed to October 2025. We recorded robotic platform availability, co-location with vascular services, robotics governance structures, and vascular representation.

**Results:**

Nine HSE Model-4 hospitals were identified, and their corresponding robotic programmes were assessed. All hospitals report access to surgical robotic platforms and institutional supports. All are co-located with vascular surgery units. No vascular robotic programmes exists in Ireland and vascular representation in national governance is simultaneously absent.

**Conclusions:**

Ireland appears ready for a centralised, mentored pilot with prospective clinical and economic evaluation. International evidence suggests feasibility with possible perioperative advantages and requires further investigation.

**Clinical trial registration:**

Not applicable.

## Introduction

Robotic-assisted surgery offers enhanced dexterity, precision, and three-dimensional visualisation across multiple surgical disciplines [1, 2]. While adoption has been widespread in urological, colorectal, and gynaecological surgery, uptake in vascular surgery remains limited. No robotic aortic procedures have been reported in Ireland to date, reflecting the absence of a local laparoscopic aortic era and a limited pool of surgeons trained in robotic aortic techniques. All Irish tertiary vascular centres are co-located with high-volume oncological services where robotic platforms are established [3]. This co-location may facilitate a safe evaluation of robotic aortic surgery, provided appropriate training, governance, and evidence generation are in place.

## Materials and methods

### Design and scope

This is an environmental scan and associated commentary on robotic vascular surgery in Ireland. No formal systematic review was undertaken. The aim was to characterise infrastructural readiness for robotic-assisted vascular surgery and the current governance/training context in Ireland.

### Data sources and centres

Publicly available sources (hospital and health-service websites, annual reports, press releases, procurement notices, specialty training programme pages, professional society materials, and newspaper articles) were reviewed up to October 2025. Centres assessed were public HSE Model-4 hospitals. No patient-level data were accessed.

### Search approach

Each institution’s website was queried using site‑specific search strings combining ‘robotic’, ‘da Vinci’, ‘robot‑assisted’, ‘vascular’, and ‘aortic’. General web searches used the same terms combined with the institution name. All sources were archived (URL/date) in a spreadsheet.

### Study selection and verification

Entries were screened for relevance (robotic programme status, specialties, platform type, year of start). Two reviewers independently verified each entry against at least one additional source or internal confirmation; disagreements were resolved by consensus.

### Data items and extraction

For each centre, we recorded: (i) presence/absence of a surgical robotic platform; (ii) Specialties using platform; (iii) apparent co-location of robotics with vascular services; (iv) publicly documented governance structures (e.g., robotics committees); and (v) whether vascular surgery was represented. Findings were summarised descriptively to inform the commentary. Author professional knowledge was used to contextualise findings but was not treated as data for centre-level counts unless a public source was available.

### Ethics

No formal ethics were required for this review of publicly available information.

## Results

### Centres identified

Nine HSE Model-4 hospitals with vascular surgery units were identified, listed in Table 1.

**Table 1:** Irish tertiary vascular centres and publicly reported robotic infrastructure

Centre	Robotic platform present	Specialties using platform (publicly listed)	Vascular co-location	Robotics committee public info	Vascular represented
St James’s Hospital [4, 5]	Yes	Thoracic; Urology; Gynaecology	Yes	Unspecified	No
Mater Misericordiae University Hospital [6]	Yes	Urology; Cardiothoracic; Colorectal; Head & Neck; Gynaecology; Hepatobiliary	Yes	Unspecified	No
Beaumont Hospital [7–9]	Yes	Upper GI; Breast; Urology	Yes	Unspecified	No
University Hospital Waterford [10]	Yes	Unspecified	Yes	Unspecified	No
University Hospital Limerick [11]	Yes	Colorectal; Urology	Yes	Unspecified	No
University Hospital Galway [12, 13]	Yes	Urology; Cardiothoracic	Yes	Unspecified	No
St Vincent’s University Hospital [14]	Yes	Multi-specialty programme. Urology; Thoracic	Yes	Yes	No
Cork University Hospital [15]	Yes	Urology	Yes	Unspecified	No
Tallaght University Hospital [16]	Yes	General Surgery; Urology; Gynaecology	Yes	Unspecified	No

### Robotic platform access

All nine centres are co-located with services that publicly report access to surgical robotic platforms via oncologic and/or thoracic programmes.

### Institutional supports (publicly reported)

The level of detail is inconsistent across sites. Multiple centres are vague with regards coordinator roles, maintenance arrangements, and local governance structures for robotic surgery.

### Vascular representation

A national framework for robotic surgery governance exists [17]. Vascular surgery is not explicitly listed among represented specialties. Centre-level documents describing vascular-specific robotic credentialing/training were not identified.

## Discussion

The co-location of vascular services with established robotic programmes suggests infrastructural readiness in all Irish Model-4 hospitals, including access to platforms and existing institutional supports [3]. These shared systems already have trained robotic coordinators, maintenance infrastructure, and procedural governance, substantially lowering the logistical barrier to initiating robotic vascular programmes [18]. The key limitation, therefore, lies not in resources or infrastructure. The principal constraints relate to surgeon training in robotic-assisted vascular procedures, such as aorto-iliac interventions, and the absence of vascular-specific pathways for credentialing. No public documentation of vascular specific robotic programmes exist, and the authors are also in possession of knowledge that no robotic-assisted vascular procedures have been performed in Irish Model-4 hospitals.

For robotic aortic surgery to progress beyond theoretical feasibility, advocacy at a national governance level is essential. A Royal College of Surgeons in Ireland (RCSI) lead governance structure to oversee robotic surgery implementation nationally was created: *‘Robotic Surgery Governance in Ireland: A guide to good practice’* [17]. The framework included representatives from general surgery, urology, ENT, cardiothoracic surgery, orthopaedic surgery, and gynaecology, but notably, vascular surgery was not represented. This omission underscores the current state of vascular surgery within Ireland’s robotic development strategy and echoes the global lack of robotic aortic interest and consequent skills development [19]. Without dedicated representation, the vascular specialty lacks both a policy voice and strategic inclusion in training, infrastructure allocation, and future robotic expansion.

To ensure equitable access to innovation, vascular surgery must be formally integrated into national robotic governance structures. Advocacy from the Irish Vascular Society, National Clinical Programme in Surgery (NCPS), and relevant academic partners will be critical to achieving this. A coordinated, multidisciplinary effort, combining governance inclusion, training development, and outcome auditing, is necessary if robotic vascular surgery is to move from theoretical possibility to clinical reality.

Bespoke training opportunities and new skill acquisition will therefore be paramount to the success of an unique Irish robotic-assisted vascular programme. Developments should include collaboration with European centres experienced in robotic aortic surgery, structured proctorship, and competency-based curricula tailored to robotics rather than adapted from historical laparoscopy [20–23]. National aortic case volumes are modest in Ireland and distributed across multiple sites. Concentrating initial robotic aortic activity in one to two centres would support team learning, quality assurance, and alignment with volume–outcome principles [24]. 

Although platform access already exists, procedure-level costs and staffing requirements may exceed those for conventional open repair [18, 25]. A prospective health-economic evaluation, integrated from the outset of any pilot, is necessary to determine value relative to open and endovascular options.

### Limitations

This environmental scan relied primarily on publicly available information, which may not represent operational realities and can under-report active programmes. For example, the authors are aware of ongoing robotic activity in gynaecology, urology, and colorectal surgery at University Hospital Waterford that is not fully documented online. To avoid unverifiable centre-level misclassification, such author knowledge was not counted in descriptive tallies. There is, in the absence of documentation to the contrary, no vascular robotic surgery programme in Ireland at present.

### Opportunity versus evidence

Ireland stands at a crossroads between readiness and restraint. The absence of prior laparoscopic experience may prevent a natural bridge to robotic-assisted surgery. However, viewed from an alternative perspective, it may represent an opportunity to adopt robotic technology without legacy bias. The infrastructure exists, but national vascular training curricula and governance frameworks have not yet integrated robotic competencies. The recently published ‘*Vascular Surgery: A model of care for Ireland’* framework by the NCPS did not reference robotic surgery, again, highlighting a lack of internal desire within the Irish vascular network to pursue this endeavour [26].

### Should Ireland invest now?

Perhaps cautiously, but strategically. A structured national feasibility programme, incorporating international mentorship and outcome auditing, would allow Ireland to safely evaluate this technology while contributing to the global evidence base. Such a programme would also align with Ireland’s strategic emphasis on clinical innovation and surgical research translation.

However, sustained progress will depend on national advocacy. Without formal representation within robotic surgery governance, vascular surgery risks being excluded from Ireland’s next phase of surgical innovation. This representation needs to come from within the current Irish vascular organisations if change is going to occur. Only through coordinated advocacy and inclusion can the potential for robotic aortic surgery in Ireland be realised.

## Conclusion

Ireland appears infrastructurally positioned to evaluate robotic-assisted vascular surgery through a centralised, feasibility programme requiring proctorship and robust health-economic analysis. Broader adoption should await comparative effectiveness data and demonstration of sustainable value. Ireland presents itself as an unique location to contribute to the ongoing investigation into robotic-assisted vascular surgery, providing desire for implementation and national advocacy is instituted.

## Data Availability

No new datasets were generated or analysed. Information was derived from publicly available institutional sources cited in the manuscript.

## Declarations

### Permission to reproduce material from other sources

Not required. No copyrighted figures, tables, or images were reproduced.
